# Maternal Impressions on Fœtus in Utero

**Published:** 1889-12

**Authors:** A. N. Denton

**Affiliations:** Austin, Texas


DANIEL'S

### TEXAS MEDICAL JOURNAL.

A Representative Organ of the Medical Profession, and an Exponent of Rational Medicine; devoted to the Organization, Advancement and Elevation of the Profession in Texas.

PUBLISHED MONTHLY.—SUBSCRIPTION $2.00 A YEAR.

Vol. V. AUSTIN, DECEMBER, 1889. No. 6.

### ORIGINAL ARTICLES.

CONTRIBUTED EXCLUSIVELY TO THIS JOURNAL.

*The Articles in this Department are accepted and published with the understanding that we are not responsible for, nor do we indorse the views and opinions of the writers by so doing.*

For Daniel's Texas Medical Journal.

### CHANGES UPON THE BODY OF THE FŒTUS BY MENTAL IMPRESSIONS.

BY A. N. DENTON, M. D., AUSTIN, TEXAS.

[Read Before the Austin District Medical Society, September 20, 1889.]

THOSE who adopt the materialistic views of Darwin and Haeckel can never satisfactorially account for the physical changes which are so often effected upon the body of the fœtus in utero by mental impressions more or less profound upon the mind of the mother.

That such changes and physical modifications are often effected in this way can scarcely be questioned by any attentive reader of the various works on physiology and midwifery. In a paper of this scope, I can only cite you to a few examples in proof of the assertion that these impressions are often followed by such physical changes, and even death of the fœtus in utero.

Montgomery in his admirable work on pregnancy, alludes to and quotes a case reported by Morgagne, in which a mental impression was quickly followed by the death of the fœtus.

Montgomery says: "The question whether mental emotions do influence the development of the embryo must be answered in the affirmative. Instances have undoubtedly occurred of such material impressions; fright, more particularly when violent, giving rise to malformation. An instance of this kind occurred under my own observation several years ago, so remarkable that I trust I shall be excused if I think it presents something more than a mere striking coincidence.

"A lady pregnant for the first time, to whom I recommended frequent exercise in the open air, declined going out as often as was thought necessary, assigning as her reason, that she was afraid of seeing a man whose appearance had greatly shocked and disgusted her; he used to crawl along on the flagway on his hands and knees with his feet turned up behind him, which latter were malformed and imperfect, appearing as if they had been cut off at the instep, and he exhibited them thus, and uncovered, in order to excite commiseration. I afterward attended this lady in her confinement, and her child, which was born a month before its time, and lived but a few minutes, although in every other respect perfect, had the feet malformed and defective precisely in the same way as the cripple who had alarmed her, and whom I had often seen.

"Still more recently, I witnessed the following fact: Mrs. N., the wife of a clergyman, came to town for her confinement, and a lady who was with her told me that she had been very uneasy in her mind from an apprehension that her child would be born with a deformed hand; her anxiety had been induced by the following occurrence: The mistress of a school which she frequently visited, had been delivered of a child with a deformed hand, and as Mrs. N. was known to be at all times very nervous and easily alarmed, and was then only a short time pregnant, great pains were taken to prevent her seeing the child, except with such precaution as would preclude her observing the hand; it happened, however, one day that she walked unexpectedly into the room where it lay asleep, and sat down by the cradle to look at the child, which at the moment happened, unfortunately, to have the deformed hand fully exposed to view. She felt greatly shocked, and often afterward alluded to what she had seen, and expressed her conviction that her child would be born with a similar deformity. Very soon after her delivery, she expressed an anxious wish to see her infant, which was brought to her wrapped up in a flannel in the usual way. She instantly drew out the child's arm, and with a look of horror exclaimed: 'Oh the dreadful hand!' and there it certainly was, with exactly the same deformity as that which had excited her disgust and terror several months before. The deformity consisted in the absence of one finger, and the complete union of the middle and third fingers, the united extremities of which were covered by one nail, presenting a very disagreeable appearance indeed."

"Several instances have come to my knowledge in which impressions from external objects have produced in the lower animal, also, corresponding effects on their offspring."

Dr. Dunglison, in his medical dictionary, in his enumeration of the causes of monsters, gives the first place to "the influence of the maternal imagination on the fœtus in utero." But I will not consume your time by further quotations, some of which might be adduced to the same effect from many of the leading authors of this and past generations.

The truth is that this principle has long been known, and recognized, not only by the medical profession, but by breeders of stock, who have practically demonstrated the truth of the principle in the modification of color and other characteristics of horses, cattle, etc. A very striking instance of the turning of such knowledge to good account in a financial point of view, is shown in the sharp practice of the patriarch Jacob in his dealings with his father-in-law, Laban.

In the contract between them, it was agreed that Jacob should have for his share all the increase of the cattle, except those of uniform color, but by a peculiar device of placing rods of variagated colors at the watering troughs, where they were seen by the kine at the moment of conception, the color of the increase were so modified as to inure greatly to Jacob's advantage, so that he became rich, while his father-in-law became poorer. But I deem it unnecessary to consume your time by further argument in support of the proposition that these impressions are made, as these effects on the fœtus have so often been witnessed and recorded by careful and truthful observers, that the proof of the proposition amounts to a demonstration.

Therefore, the only remaining question of interest in connection with the subject under discussion is, how these impressions are made; through what medium are such results accomplished. Evidently, to my mind, through the mental organism of the mother. The truth is, it is impossible for these impressions to be made through any other medium, and those who adopt the views of Messrs. Haeckel, Huxley and Tyndal are totally unable to account for this and many other phenomena in connection with man's existence, upon any physical hypothesis.

Some months ago, in a paper which I had the honor of reading before this society, I endeavored to prove, and I think I did prove, to the satisfaction of the unprejudiced, the duality of man's existence, as well as that of every living creature.

Since that time rather extensive reading and reflection upon this subject has only strengthened my convictions in this direction, and in reaching this conclusion I have not in any degree been biased or influenced by the tenets taught by Christianity or the Bible. The same questions which I then discussed are involved in the subject of maternal impressions on the human embryo or fœtus in utero, and, while I do not intend to weary you, by traveling over the same ground, I desire to submit for your consideration some additional thoughts upon the subject.

While I am ready to record my admission that the exact relations existing between man's physical system and his mental organism, as well as to the consequences to the latter, of the death and disintegration of the former, are at present to me unknown and inexplicable, I claim that the existence of this mental entity of man can be fairly demonstrated by a variety of phenomena seen in everyday life, and that without the admission of the existence of this living intelligence, this substantial though intangible entity, many of these phenomena are wholy inexplicable that admit of easy explanation upon this hypothesis. It is a well known fact that physical as well as mental characteristics are transmitted from parent to child. Diseases in great variety are also inherited from generation to generation, as is generally supposed through or by the corporeal structure of the body. But if we admit the truth of the well known theory of disintegration, waste and supply of the physical system, as taught in every standard work on physiology, we are compelled to reject the doctrine of transmission of physical or mental characteristics through the medium of the blood or other part of the corporeal structure. It is impossible that the physical body can be the medium of such transmission; because every atom composing it is disintegrated and removed from the system, and new material substituted several times during the average period of an ordinary human life. This fact cannot be controverted, nor will it be disputed. I ask then, how is it possible that the corporeal body can be the medium or agent for such transmission? And if the physical system is not the medium through which these characteristics are inherited, then by exclusion, it must be admitted that such transmission is effected through that intangible entity, which we call mind. Memory, too, is wholly inexplicable upcn any other theory than the one assumed.

Nothing can be more absurd or unreasonable, than to suppose that the events of the past are permanently retained upon the corporeal structure of our bodies, while it is universally admitted that the entire mass of this structure is disintegrated and washed away several times during the course of our lives.

It would be as rational and reasonable to write the statutes of your State upon the ever shifting sands which are perpetually washed by the waves of the gulf of Mexico, and expect it to remain a permanent record, as to expect those impressions to be indefinitely retained upon a structure that is forever undergoing changes which amounts to frequent and complete destruction. Surely the admission must be made by every reasonable creature that the great store house of past events which we call memory, must have a more permanent and enduring canvas, upon which to preserve the record of those impressions, than a structure which is constantly undergoing destructive changes and mutations.

I am aware that it is objected to by materialists that the mind is often impaired or apparently obliterated by injury or disease of certain portions of the corporeal structure, and this is seized upon as an argument that it is only a property of matter and must cease to exist upon the destruction of our physical bodies.

As I have admitted in the earlier pages of this paper, there are some questions in reference to the relations existing between mind and matter that in the present state of our knowledge are inexplicable; but it may be assumed with reason that during the mysterious relationship which exists between our mental and physical organisms, the former would not usually be affected and modified in various ways by disease or destruction of parts of the latter during their association.

Striking analogies in support of this assumption may be seen in the generation and transmission of the electric fluid.

But I have neither time nor disposition to discuss these intricate questions in detail. My motive in the outset was only to prove the existence within our physical bodies of a more permanent and enduring medium, through and by which these maternal impressions are made on the fœtus in utero.

Many additional proofs of the existence and ceaseless activity of this vital organism might be adduced; among the number the healing of wounds of any kind and grade, and the efforts of this vital intelligence to restore the parts and organs to their original shape and symmetry, affords numberless striking examples and proofs of the duality of every living creature.

A still more impressive proof of such dual nature is afforded in the complete reproduction of the tail of the Salamander, after its detachment from the body of the animal. During my boyhood I have often detached or cut off the tails of the common brown lizzard and have often witnessed their complete reproduction in the exact form of the original organ.

Can physicists who hold to the materialistic views of Messrs. Haeckel and Huxley, satisfactorily account for this curious phenomenon? How is it possible for philosophers who exclude all living intelligence from the creation and perpetuation of life in all its forms, to account for this building and reconstruction of an entire organ?

The reproduction of the tail of this little animal presents for our consideration something more than the healing of a wound. As before stated, it is the reforming and building up of an entire organ in the exact shape of the original. How is this to be accounted for? Who is the architect of this curious reconstruction of an organ? Do the cells and molecules which compose the blood of this little animal possess the inherent intelligence to superintend the building of this new structure, and move forward in the stream of blood and climb into the proper positions, in order to reconstruct the tail of this living creature?

The truth is, the exact pattern for this piece of architecture exists in the vital organism of the animal, and by this model the process of reconstruction, its physical counterpart, is begun and carried on to completion.

But I desist at present from further elaboration of this subject, and return but for a moment to the more direct discussion of the point at issue, viz.: the medium by and through which maternal impressions on the fœtus are made. Physiological investigations have established the fact that the circulation of the fœtus in utero is distinct from that of the mother, and that the blood of the former is oxydized and its physical system supported and nourished by the exposure of its blood through the medium of the placenta to the arterial blood of the mother, the process being somewhat similar to that seen in the beings after birth.

Neither have physiologists been able to trace any direct venous communication between mother and fœtus. Thus it seems that so far as any direct physical communication between the two is concerned, the circulatory and nervous systems are upon an equal footing. But I think it will scarcely be seriously questioned that, notwithstanding the fact that scientists have hitherto failed to discover the physical conductors through which such influences are usually transmitted, there does exist an intimate nervous relationship between the mother and fœtus, though perhaps in a modified form.

There is, I think, too great a disposition to discredit the existence of all substance, save that which can be recognized by our senses, and hence, because the mental entity which we call mind, cannot be seen or felt, its very existence is questioned or disputed, although the results of its operations and its ceaseless activity are plainly visible on every hand.

From what has been said in the preceding pages, I think the following conclusions may be legitimately drawn:

1. That profound impressions through the mother involving physical changes on the fœtus in utero, though rare, have been frequently observed and cannot reasonably be questioned.

2. That such impressions sometimes profoundly change and modify the physical and mental organism of the embryo or fœtus, even to the extent of great physical deformity in some instances.

3. That these impressions are made through the mental organism of the mother.

